# Predicting cardiovascular outcomes in elderly patients with acute coronary syndrome: a nomogram approach

**DOI:** 10.21203/rs.3.rs-8058920/v1

**Published:** 2025-12-17

**Authors:** Hamidreza Soleimani, Reza Nikfar, Sahand Siami, Farhad Shaker, Parisa Fallahtafti, Mehdi Mehrani, Yaser Jenab, Sajjad Hosseini, Adrian V. Hernandez, Diaa Hakim, Michael G. Nanna, Kaveh Hosseini

**Affiliations:** Imam Khomeini Hospital Complex Tehran University of Medical Science; Tehran Heart Center Research Institute, Tehran University of Medical Sciences; Tehran Heart Center Research Institute, Tehran University of Medical Sciences; Tehran Heart Center Research Institute, Tehran University of Medical Sciences; Tehran Heart Center Research Institute, Tehran University of Medical Sciences; Tehran Heart Center Research Institute, Tehran University of Medical Sciences; Tehran Heart Center Research Institute, Tehran University of Medical Sciences; Imam Khomeini Hospital Complex Tehran University of Medical Science; University of Connecticut School of Pharmacy; Harvard Medical School; Yale School of Medicine; Tehran Heart Center Research Institute, Tehran University of Medical Sciences

**Keywords:** MACE, STEMI, PCI, Model Prediction

## Abstract

**Background:**

Although ST Elevation Myocardial Infarction (STEMI) diagnosis and therapy have improved, high-risk categories like elderly persons still have a significant chance of MACE despite treatment.

**Objectives:**

This study attempts to construct a predictive nomogram for MACE incidence using clinical data from a STEMI registry.

**Methods:**

Tehran Heart Center’s computerized record recognized all 65-year-old STEMI primary PCI patients consecutively. This retrospective study examined demographic, laboratory, clinical, and intra-procedural factors. Post-PCI univariate and multivariate analyses identified MACE risk variables. Decision curve analysis, ROC, and calibration plots validated predictive nomograms. R Studio and R used “tidyverse” and “rms” packages for all analyses.

**Results:**

The 1946 study included 70% training and 30% testing patients. Basic demographic and clinical variables were identical for both groups. The average follow-up was 17 months. 8 factors were selected for the nomogram after univariate and multivariate analysis: left-ventricular ejection fraction (LVEF), serum creatinine, hemoglobin, and fasting blood glucose levels, presence of valvular heart disease, post-PCI TIMI flow grade, diameter of the culprit lesion stent, and presence or absence of shock after PCI. The post-PCI MACE prediction AUC was 71%. Calibration plots showed that the nomogram model was well-calibrated and close to observed outcomes. Decision curve analysis also revealed that the model predicted MACE discriminatively.

**Conclusion:**

A nomogram successfully predicts MACE risk in older STEMI patients using laboratory, clinical, and procedural parameters. This algorithm may identify vulnerable high-risk patients for more aggressive preventative interventions.

**Clinical trial number:**

not applicable.

## Introduction

Ischemic heart diseases (IHDs), including acute coronary syndrome (ACS), are the leading cause of death worldwide, accounting for approximately 9 million deaths in 2021 [1]. Advanced age is one of the most important predictors of ACS morbidity and mortality one year following hospitalization [2, 3]. The vast majority of elderly patients are typically undertreated compared to younger patients following ACS. Evidence indicates that younger patients with ACS undergo emergency reperfusion three times more frequently than their older counterparts, and antithrombotic medications, diuretics, and statins are significantly underused after older patients are discharged [3, 4].

Recently, in various countries, efforts have been made to decrease in-hospital mortality and improve the incidence of adverse outcomes following ACS by applying evidence-based ACS therapies, early invasive surgery, and efficient post-percutaneous coronary intervention (PCI) care [4]. Also, evaluation and resolution of comorbidities and geriatric syndromes, including frailty, altered mental status, and cognitive disorders, and incorporating them into risk assessment models such as the combined model of GRACE risk score and clinical frailty scale (CFS) in elderly patients, can dramatically reduce in-hospital and long-term mortality after ACS [5–8].

A plethora of risk scores have been developed to predict poor outcomes and mortality in patients who have had ACS. However, few risk assessment tools have gained extensive credibility and validation to measure the risk score in patients following ST-elevation myocardial infarction (STEMI). Moreover, most studies have built their risk prediction models without focusing on the elderly population specifically. Some studies suggest that, despite their advantages in calculating the risk score prior to coronary angiography, prediction models merely based on clinical variables may miss the anatomical parameters that can increase discrimination and calibration for predicting adverse events. Therefore, A combined score model of clinical and angiographic variables, such as the Clinical SYNTAX Score (CSS), can significantly boost the model’s discriminative ability [9]. Other recent cohort studies have concluded that a nomogram-based prediction tool that includes both clinical and anatomical factors can stratify patients based on risk association and improve clinical decision-making by predicting major adverse cardiovascular events (MACE) incidence following ACS in the elderly [10].

Since there are only a limited number of reliable and validated risk stratification tools assessing MACE probability in STEMI patients over 65, in this retrospective registry study, we aimed to develop a precise and actionable nomogram with good calibration and discriminative ability to accurately predict MACE incidence in these patients.

## Methods

### Study population

This study included patients aged 65 years and above diagnosed with STEMI and undergoing primary PCI at Tehran Heart Center. Clinical, biochemical, and procedural data for this research were meticulously extracted from the institutional database, which contains detailed patient records spanning from 2015 to 2020. A total of 1,946 patients who met the inclusion criteria were identified. To maintain consistent outcome distribution, the dataset was stratified by MACE incidence and these patients were stratified into a 70:30 ratio for training and testing purposes, resulting in 1,362 patients in the training set and 584 patients in the testing set. Institutional Review Board (IRB) and ethics committee at THC approved this study (IR.TUMS.THC.REC.1399.045). This study was retrospective in nature, utilizing de-identified data from THC’s electronic registry, so an informed consent waiver was granted by the IRB.

### Biochemical tests

Before emergency coronary angiography, blood samples were analyzed to assess fasting blood glucose (FBS), hemoglobin (Hb), and serum creatinine levels. Lipid profile measurements, including total cholesterol (TC), triglycerides (TG), low-density lipoprotein cholesterol (LDL), high-density lipoprotein cholesterol (HDL), the TG/HDL ratio, and the triglyceride-glucose (Tyg) index, were retrieved from the database. The Tyg index, a proxy for insulin resistance, was calculated using the formula: [(Fasting triglycerides (mg/dL) × Fasting glucose (mg/dL)) / 2].

### Coronary angiography

Upon hospital admission, all patients underwent coronary angiography performed by senior cardiologists following standard treatment protocols. Procedural details, including pre-procedure administration of guideline-recommended loading doses of Ticagrelor/Clopidogrel and Aspirin, were documented in the database. Following PCI, patients were prescribed standard treatment regimens, including moderate-intensity statins and modern antiplatelet therapies. Follow-up schedules and treatment adherence were recorded as part of the institutional database.

### Follow-up and outcomes

Outcomes were monitored through clinic visits and phone interviews, with all relevant data systematically logged in the database. Major adverse cardiac events (MACEs) were defined as a composite outcome encompassing all-cause mortality, nonfatal myocardial infarction, and stroke.

### Statistical analysis

Variables with less than 10% missing data were selected from the database, resulting in 38 clinical, biochemical, and procedural factors included in the analysis. Variables with > 10% missingness were excluded; these were primarily procedural factors (e.g., stent brand, inflation pressure, operator name) without established prognostic relevance. For all other variables (< 10% missingness), missing values were managed using multiple imputation by chained equations under a missing-at-random assumption. Statistical analysis was done using R Studio version 1.1.463 (Posit PBC, Boston, MA, United States), utilizing packages *tidyverse* [11], *pROC, survival*, and *rms*. Means and standard deviations (SDs) were presented for continuous variables with normal distributions and medians and interquartile ranges (IQRs) for continuous variables with skewed distributions. For the analysis of continuous variables with normal distributions, the t-test was utilized. For the analysis of continuous variables with nonnormal distributions, the Mann-Whitney U-test or the Kruskal-Wallis test was utilized. Analysis of categorical variables was carried out utilizing the x2 -test. In all comparisons, a p-value below 0.05 indicated statistical significance.

In the training set, a comprehensive univariate analysis was performed in this step, ensuring that all variables that were independently associated with MACE incidence and were statistically significant were selected. All variables were analyzed through a multivariate model to evaluate their association with MACE, with significance determined by a p-value threshold of 0.05. The proportional hazards assumption was evaluated using Schoenfeld residuals and log-minus-log plots, with no significant violations observed. The Cox model was specified to include main effects only, as interaction terms were not formally modeled to preserve parsimony and avoid unstable estimates. These predictors were subsequently used to construct a predictive nomogram for estimating MACE risk. R software was utilized to construct the nomogram using the Regression Modeling Strategies (*rms*) package. Risk nomograms were analyzed utilizing receiver characteristic curves (ROC) to evaluate their ability to discriminate MACEs based on the calculation of area under the ROC curve [12]. Utilizing the *rms* package, calibration curves were plotted and calculated to evaluate the calibration of the MACEs after the construction of the ACS risk nomograms. Bootstrapping was carried out by repeated random sampling (1000 times) for internal validation of model accuracy. The resulting optimism-corrected estimates were then used for Decision curve analysis (DCA) to quantify the clinical efficacy of the nomograms based on their net benefit for different threshold probabilities [12].

## Results

### Population characteristics

A total of 1946 elderly STEMI patients who underwent PCI were included in this study, with a mean follow-up period of 17.68 ± 14.50 months. The mean age of this population was 73.38 ± 6.99 years, and 68% were male. This population was divided into a train set (70% of the total population, 1362 cases) and a test set (30% of the total population, 584 cases). These two groups were statistically comparable regarding all investigated demographic, laboratory, clinical, and procedural characteristics except for the door-to-needle interval, which was longer in the train set (**Supplemental Table 1**). Among the total population, the overall incidence of MACE was 18.29%, with a comparable rate in the two train and test sets.

### Probable predictors of MACE in the elderly with STEMI

A stepwise method using univariate and multivariate Cox regression was employed to determine the independent predictor of MACE in elderly STEMI cases undergoing PCI (**Supplemental Table 2**). In multivariate model 8 factors were identified to be significantly independently associated with MACE incidence: LVEF (OR = 0.96, 95%-CI: 0.94–0.98), serum creatinine (OR = 1.41, 95%-CI: 1.23–2.33), hemoglobin (OR = 1.01, 95%-CI: 1–1.01), fasting blood glucose levels (OR = 0.88, 95%-CI: 0.81–0.95), presence of valvular heart disease (OR = 2.39, 95%-CI: 1.24–5.87), post-PCI TIMI flow grade < 2 (OR = 0.43, 95%-CI: 0.19–1), diameter of the stent placed in the culprit lesion (OR = 0.69, 95%-CI: 0.49–0.98), and the presence or absence of shock in the post-PCI setting (OR = 28.4, 95%-CI: 9.45–123). Additionally, the overall incidence of MACE in the study cohort was 18.3%.

### Construction of a nomogram predicting MACE following STEMI

Using the results of multivariate analysis and employing the eight identified independent predictive factors, a nomogram was constructed to predict the risk of MACE following STEMI in the elderly undergoing PCI (*Central illustration*).

Drawing a vertical line from the value of each predictive factor to the “points” line could determine its numerical points. The total point could be calculated by summing up all of these points, representing the patient risk for MACE (ranging from 0 to 100). To assess the probability of MACE incidence, a vertical line from the calculated total point on the “Total points” line could be drawn to the “Risk of MACE incidence” scale on the bottom of the nomogram.

### Nomogram validation and predictive performance

**Figure 1:**
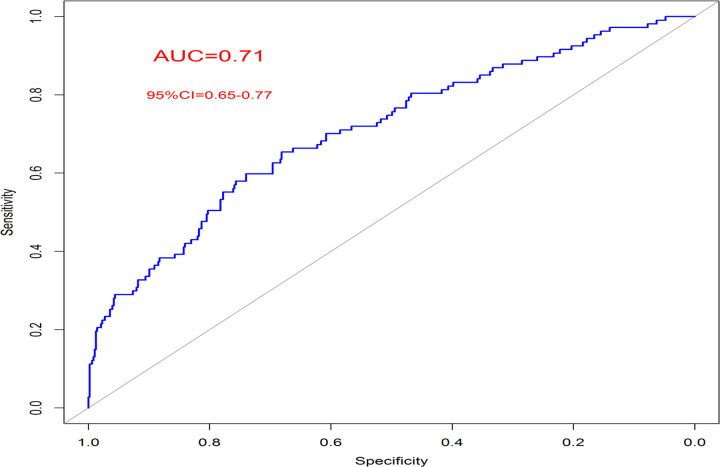
ROC analysis of the accuracy of the nomogram in predicting MACE incidence in the development cohort.

To assess the discriminatory ability of our nomogram, receiver operating characteristic (ROC) analysis was performed, which illustrated an area under cover (AUC) of 0.71 (95% CI: 0.65–0.77) (Fig. 1). At the optimal cutoff determined by Youden’s index, the model achieved a sensitivity of 72%, specificity of 63%, positive predictive value of 30%, and negative predictive value of 91%.

**Figure 2:**
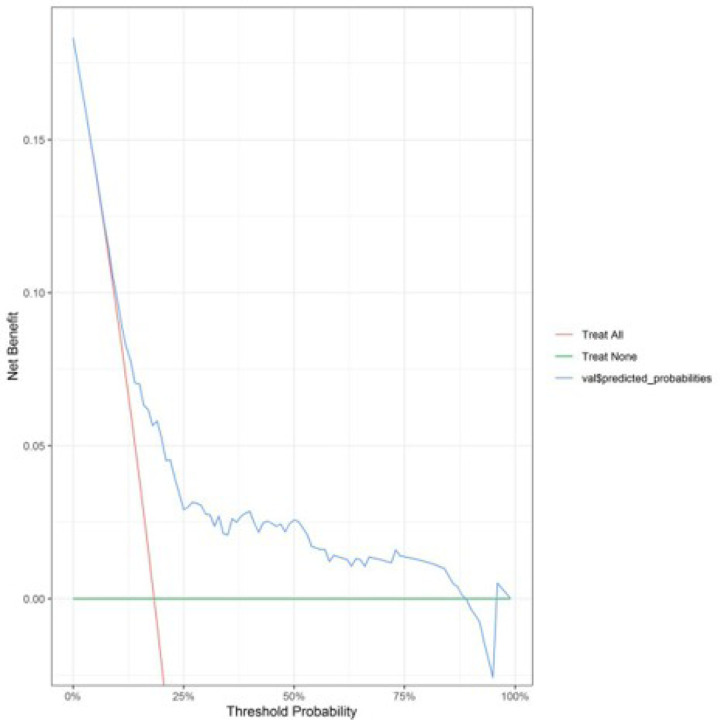
Decision curve analysis (DCA) was conducted to predict the incidence of MACE in the development cohort. The plot illustrates the correlation between threshold probability (abscissa) and the net benefit (ordinate) of the prediction model (blue line), the scenario of treating all patients (red line), and the scenario of treating none of the patients (green line).

A decision curve analysis (DCA) was performed based on optimism-corrected estimates to determine the net benefit of our developed prognostic model for clinical decision-making, which demonstrated a threshold probability of 23 to 89% for MACE incidence following PCI in patients > 65 years of age (Fig. 2), which suggests a wide range of applications. Additionally, a calibration plot was also generated to investigate the similarity and concordance of our nomogram’s predicted probability with the observed probability.

**Figure 3:**
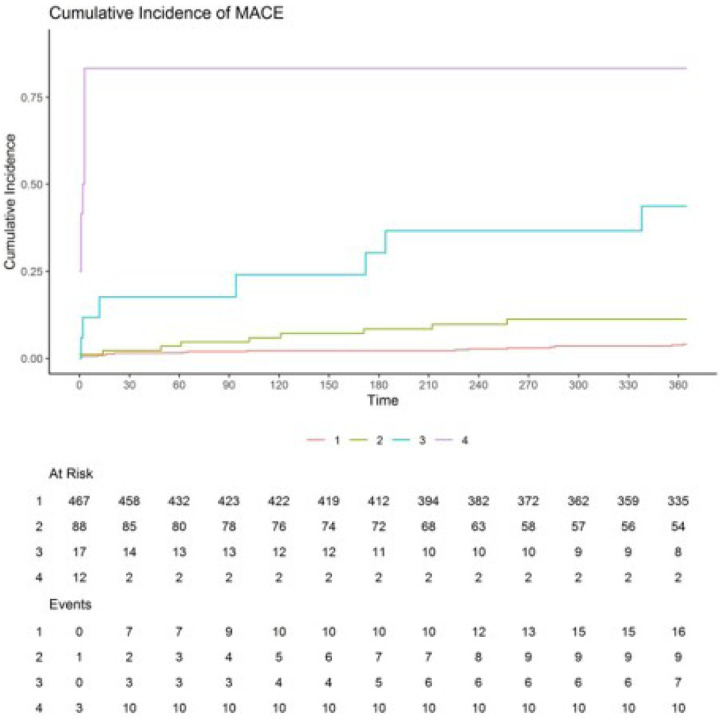
The incidence of cumulative MACE among patients categorized by different prognostic index quartiles.

In addition, Kaplan-Meier analysis was performed to investigate the prognostic reliability of the nomogram’s predicted risk of MACE. We stratified our patients into four risk groups according to the nomogram’s predicted risk (0–0.25 as low risk, 0.25–0.5 as intermediate risk, 0.5–0.75 as high risk, 0.75–1 as very high-risk group). The results of the Kaplan-Meier analysis have been illustrated in the training set (Fig. 3-A), test set (Fig. 3-B), and total population (Fig. 3-C).

According to our results, very high-risk patients (predicted risk of > 0.75) demonstrated a 13.12 (95%-CI: 7.09–24.25) and 16.74 (95%-CI: 8.73–32.1) hazard ratio of MACE incidence compared to all other cases (predicted risk of < 0.75) and low-risk cases (predicted risk of < 0.25), respectively. A similar trend of higher observed MACE incidence in groups with higher nomogram’s predicted risk was observed through all other possible inter-group comparisons (**Supplemental Table 3**), which suggests the clinical reliability of our nomogram’s predicted risk of MACE incidence.

A mean absolute error of 0.018 was calculated for our model’s calibration plot, indicating that, on average, the predicted probabilities deviate from the actual outcomes by 1.8% (**Supplemental Fig. 1**). The precision recall curve indicates excellent precision across various recall levels with an AUC-PR of 0.9 (**Supplemental Fig. 2**).

## Discussion

Our study developed and validated a nomogram for predicting MACE in a cohort of 1,946 elderly patients who underwent primary PCI. The nomogram, based on 8 significant predictors, was assessed for its discriminatory ability using ROC analysis, which yielded an AUC of 0.71. Kaplan-Meier analysis further evaluated its prognostic reliability by stratifying patients into four risk groups based on predicted MACE risk. This analysis, illustrated in both the train and test sets, revealed that very high-risk patients (predicted risk > 0.75) had significantly higher hazard ratios for MACE compared to lower-risk groups, affirming the nomogram’s clinical reliability.

Effective prognostic information is often obtained through scoring systems. The TIMI risk score is a straightforward tool originally designed to predict 30-day mortality in STEMI patients treated with fibrinolytics, and it has also proven effective for predicting in-hospital mortality in STEMI patients undergoing primary PCI [13, 14]. The Controlled Abciximab and Device Investigation to Lower Late Angioplasty Complications (CADILLAC) risk score was developed to predict both 30-day and one-year mortality in STEMI and NSTEMI patients following primary PCI [15]. Compared with the mentioned and other traditional risk scores, including TIMI, PAMI, CADILLAC, and GRACE, which offer a modest discrimination in both the general STEMI (AUCs ranging from 0.68 to 0.70) [16] and elderly population, with an AUC of 0.70 and 0.69 for in-hospital and post-discharge mortality, respectively [17] our nomogram model demonstrates improved predictive power in the patient population aged ≥ 65 years, a population being rarely investigated in the existing literature. [18, 19]

The incorporation of the most robust clinical predictors for the elderly population following PCI, including age, diabetes, prior MI, LVEF, STEMI presentation, and eGFR, which were identified by Jalali et al.’s meta-analysis [20], alongside real-time procedural characteristics like post-PCI TIMI flow, stent diameter, and post-PCI shock, into our nomogram, has led to the development of a more bedside-friendly and homogeneous risk assessment tool for clinicians and patients, as other machine learning models such as iPROMPT [21] and XGBoost with a large sample size [22], rely more on heterogeneous predictors and “black-box” algorithms while showing more discriminative ability.

Zhu et al. [18] have developed a similar nomogram in the elderly population post-PCI, but lacked the distinction between STEMI and NSTEMI populations, underestimating the possible clinical discernibility between these two. Although more discriminative ability was achieved by Zhu’s model with an AUC of 0.787, the high reliance of this model on biomarkers, overlooking the mechanical and hemodynamic dimensions of risk, which are addressed by procedural variables, and a smaller sample size of nearly 1000 patients compared with our nomogram, demonstrates the high clinical utility of the current model. Unlike our model, which addressed the risk of MACE following PCI in a specific age group, Yao et al. [23] developed another risk-prediction nomogram model, incorporating six variables, for 1-year readmission due to MACE in the general STEMI patients’ population with a considerably smaller sample size, lowering the generalizability of this tool in the clinical environment.

Our model addresses some methodological shortcomings mentioned in a study that systematically explored the prediction models for MACE following PCI. With the inclusion of nearly 2,000 patients aged ≥ 65, we minimized heterogeneity and increased applicability for the elderly population while providing an events-per-variable (EPV) above the ≥ 20 threshold. The incorporation of real-time procedural characteristics into our model alongside the implementation of multivariable analysis addressed the lack of multivariable modeling for the selection of MACE predictors and the underestimation of procedural variables for risk stratification, as mentioned by Deng et al. [19] Moreover, we presented both calibration plots and Hosmer-Lemeshow statistics, directly addressing what 74% of models failed to report in Deng’s review. [19]

We have enabled the risk stratification of elderly patients into four distinct risk groups based on the model’s predicted risk, which can inform individual care by directing high-risk patients to more intensive treatment plans, including earlier referral to specialized cardiology and heart failure clinics as well as extended monitoring and more intensive post-discharge follow-up. DCA is conducted to evaluate the clinical value of a prognostic model by quantifying the net benefit across a range of risk thresholds for developing a certain outcome, at which a clinician might choose to intervene. In our model, DCA delivers a positive net clinical benefit in a broad range of risk thresholds (23–89%), which translates into the applicability of the current model for both clinicians who only treat extremely high-risk patients and those who approach patients more conservatively in real-world settings. Chen et al.’s iPROMPT [21] shows a positive net benefit in a much narrower threshold range of 30–60% and Zhu’s model [18] demonstrates net benefit only when the threshold exceeds 15% which does not clarify how their net gain changes beyond moderate thresholds. Hamilton et al. [22] did not include a DCA, leaving clinicians and patients uncertain about whether this model leads to better clinical decision support in real-world settings. Fang et al.’s [24] DCA showed net benefit in a wide threshold range of 10–99% in their model, but included only 466 STEMI patients.

Importantly, although we have not yet implemented our nomogram in the institution’s electronic medical record (EMR), the model is designed for integration into EMR and real-time use at the bedside. Its variables are readily available after PCI, and its point-based graphic format supports bedside decision-making and shared discussions with patients and families. This implementation plan reinforces the model’s potential utility for cardiologists and explains how we envision its applicability in daily care alongside its direct benefit to readers and clinicians.

Our nomogram was constructed based on eight key factors identified as independent predictors of MACE in elderly STEMI patients undergoing PCI. These factors include LVEF, serum creatinine, hemoglobin, FBS, presence of VHD, post-PCI TIMI flow grade less than 2, diameter of the stent placed in the culprit lesion, and the presence or absence of shock in the post-PCI setting.

LVEF, as a critical measure of cardiac function, is particularly important in the context of STEMI, as it helps to assess the severity of cardiac damage and guides treatment decisions. Previous studies have shown that low LVEF is associated with higher rates of MACE following STEMI [25–27]. VHD is both a risk and a complication of ACS, which is associated with a worse prognosis [28, 29]. Our results align with the study of Hasdai et al., in which STEMI patients with pre-existing VHD had a worse prognosis compared to those without VHD [30].

Our nomogram incorporates easily obtainable blood test parameters, including creatinine, FBS, and baseline hemoglobin levels. Various studies have shown that higher creatinine and worse kidney function are associated with higher rates of MACE after acute MI [31, 32]. Many patients with elevated creatinine levels have comorbid conditions such as diabetes, hypertension, and chronic kidney disease (CKD), which independently contribute to worse outcomes in STEMI. Moreover, renal impairment can exacerbate the inflammatory response during MI [33]. Additionally, renal dysfunction can lead to fluid overload and hypertension, further straining the heart during an acute event [34].

Furthermore, studies have shown that stress hyperglycemia is significantly associated with an increased risk of mortality in STEMI patients treated with PCI, regardless of diabetic status [35, 36]. Stress hyperglycemia triggers the production of inflammatory factors, which can exacerbate atherosclerosis through various intracellular pathways [37]. Additionally, stress hyperglycemia increases thrombogenic activity, leading to a hypercoagulable state [38]. Anemia is another strong and independent predictor of MACE in patients with ACS, showing a notable dose-response relationship [39]. It significantly reduces oxygen delivery to the myocardium beyond coronary stenoses and increases myocardial oxygen demand by requiring a higher stroke volume and heart rate to ensure sufficient systemic oxygen delivery [40]. These factors may explain the progressively poorer outcomes seen in ACS patients with lower baseline hemoglobin levels. In the study of Liu et al., a higher hemoglobin level in the anemic group was associated with a decreased risk of 1-year mortality; however, a higher hemoglobin level in the erythrocytosis group was linked to an increased risk of 1-year mortality [41].

It is well established that peri-procedural complications during PCI can significantly impact mortality [42]. In our study, we observed that the characteristics of PCI and post-PCI events play a crucial role in patient outcomes. Notably, post-PCI TIMI flow, the occurrence of post-PCI shock, and the diameter of the PCI stent were significantly meaningful factors. Previous studies have shown that achieving TIMI flow grade 3 following PCI is linked to better short-term and long-term outcomes in these patients, including a decrease in MACE and cardiac mortality [43, 44]. Although peri-procedural cardiogenic shock is reported to be low, it was linked to a significantly higher mortality [45]. Likely, in our study, post-PCI shock was the most powerful predictor of MACE following PCI. Stent diameter was another key factor in predicting MACE in this population. The benefits of stent oversizing on procedural and clinical outcomes have been documented. Specifically, small vessels treated with smaller stents experienced more adverse events [46]. Shugman et al. reported that deploying bare-metal stents in STEMI patients with infarct-related arteries measuring 3.5 mm or larger was associated with low rates of target vessel revascularization [47]. This indicates that choosing larger stents could enhance long-term outcomes.

Our study has several strengths, including the use of a large cohort of 1,946 elderly STEMI patients, which enhances the robustness and generalizability of the findings. The rigorous analytical approach, employing both univariate and multivariate methods, allowed for the accurate identification of significant predictors of MACE. The nomogram was validated with an AUC of 0.71, demonstrating its effective discriminatory ability across train and test cohorts. Additionally, the inclusion of key clinical factors such as LVEF and post-PCI TIMI flow underscores its practical relevance. The DCA further confirmed the nomogram’s broad applicability and net benefit for clinical decision-making. However, the study has limitations such as the potential lack of generalizability due to being conducted in a single center, a follow-up period that may not capture long-term outcomes, and variability in some data points between cohorts. Other relevant factors not included in the nomogram might also influence MACE risk stratification.

### Limitation

There are several important limitations to consider. Firstly, it was conducted at a single center, which may influence the generalizability of the findings to broader populations, as our results may not fully represent diverse patient demographics or different clinical settings. Additionally, the sample size of fewer than 2,000 participants, while adequate for preliminary findings, suggests that a larger cohort could enhance statistical power and improve the detection of significant differences or associations. The absence of external validation with a larger dataset indicates an opportunity for future research to strengthen the applicability of our results across various populations. Additionally, some clinically relevant parameters were not available in our dataset. These include details of antiplatelet therapy, anatomical vessel involvement (e.g., single- vs. multi-vessel disease), and post-discharge data such as attendance at cardiac rehabilitation or adherence to guideline-directed medical therapy (GDMT). While these variables are known to influence long-term outcomes, they could not be assessed in the current study. However, our model incorporates several intra-procedural predictors, including post-PCI TIMI flow, stent diameter, and post-PCI shock, which offer insight into procedural success and clinical severity. Notably, a recent systematic review of PCI-related MACE prediction models [19] identified these procedural variables as underexplored components of existing models. The inclusion of such factors in our nomogram provides added value and reflects an effort to address this methodological gap. Importantly, we recognize that important clinical parameters, including echocardiography and electrocardiogram, were not included in this investigation, and future studies that incorporate these factors may provide a more comprehensive understanding of the outcomes. Although the model demonstrated satisfactory discrimination (an AUC of 0.71, sensitivity of 72%, specificity of 63%), further improvement may be achievable through external validation in larger multicenter cohorts, incorporation of additional anatomical and post-discharge predictors (e.g., multi-vessel disease, adherence to guideline-directed medical therapy), and the application of rigorously validated machine-learning approaches. In addition, our Cox model was specified to include only main effects. Although this approach prioritizes interpretability and stability, it assumes no effect modification between predictors. Evaluation of clinically plausible interactions in larger or external datasets is an important direction for future research. Lastly, some study limitations, including the lack of external validation in multicenter studies and standardization across different systems, are potential barriers to the implementation of our nomogram model into the EMR. Assessment of clinician and care team acceptance, evaluation of workflow impact, and feasibility testing with medico-legal considerations will enable the integration of this nomogram into the EMR and real-time bedside settings. We will focus on pursuing each step to underline the practical implications of clinical applicability and to ensure the integration of this model into the EMR.

## Conclusion

In conclusion, our nomogram, validated in 1,946 elderly STEMI patients undergoing PCI, showed an AUC of 0.71 and effectively predicted MACE risk. It integrates eight key factors and demonstrated strong clinical utility, with high-risk patients having significantly higher MACE rates. This tool offers valuable insights for personalized risk assessment and management in the elderly population with STEMI.

## Supplementary Material

Supplementary Files

This is a list of supplementary files associated with this preprint. Click to download.

- SupplementalAppendix..docx

## Figures and Tables

**Figure 4:**
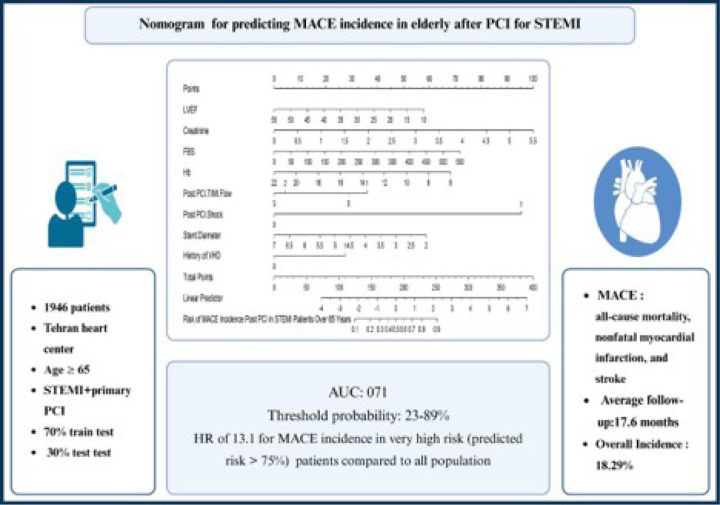
Central illustration A nomogram was developed for predicting MACE incidence using multivariate Cox regression analysis. Each patient was assigned points for each clinical characteristic by drawing a vertical line from its value to the top row. The scores for all eight characteristics were summed to calculate the total points in the middle row.

## Data Availability

The datasets used in the current study are available upon reasonable request.

## Perspectives:

### Clinical Competencies:

This study contributes primarily to the domains of Medical Knowledge, Patient Care, and Practice-Based Learning and Improvement. By developing and validating a predictive nomogram tailored to elderly patients with STEMI undergoing primary PCI, the work strengthens clinicians’ ability to identify individuals at elevated risk of post-procedural adverse events. It promotes the application of evidence-based decision-making for a high-risk. The model leverages routinely collected clinical and procedural data, enhancing bedside applicability and facilitating more personalized post-PCI management. This supports quality improvement efforts by aligning individualized care with system-wide strategies for outcome monitoring.

### Translational Outlook:

This study addresses a key translational gap by introducing a risk prediction model developed specifically for elderly patients with STEM, a population that has not been widely studied in isolation for dedicated post-PCI prognostic modeling. Most existing risk scores are derived from mixed-age cohorts, limiting their calibration and clinical relevance in older adults. By using readily available clinical and procedural variables, this model enables real-time application at the point of care without reliance on advanced diagnostics or long-term follow-up data. Future directions include external validation across broader populations, digital integration into clinical decision support systems, and incorporation of additional parameters such as anatomical burden and post-discharge adherence to optimize its performance and clinical utility.

## Abbreviations and Acronyms:

ACS: acute coronary syndrome
IHD: Ischemic heart diseases
LVEF: left-ventricular ejection fraction
PCI: percutaneous coronary intervention
MACE: major adverse cardiovascular events
DCA: Decision curve analysis
FBS: assess fasting blood glucose
TIMI: thrombolysis in myocardial infarction
